# Clinical Characteristics and Immune Responses in Children with Primary Ciliary Dyskinesia during Pneumonia Episodes: A Case–Control Study

**DOI:** 10.3390/children10111727

**Published:** 2023-10-24

**Authors:** Danli Lu, Wenhao Yang, Rui Zhang, Yan Li, Tianyu Cheng, Yue Liao, Lina Chen, Hanmin Liu

**Affiliations:** 1 Department of Pediatric Pulmonology and Immunology, West China Second University Hospital, Sichuan University, Chengdu 610000, China; 2 Key Laboratory of Birth Defects and Related Diseases of Women and Children, Sichuan University, Ministry of Education, Chengdu 610000, China; 3 NHC Key Laboratory of Chronobiology, Sichuan University, Chengdu 610000, China; 4 The Joint Laboratory for Lung Development and Related Diseases of West China Second University Hospital, School of Life Sciences of Fudan University, West China Institute of Women and Children’s Health, West China Second University Hospital, Sichuan University, Chengdu 610000, China; 5 Sichuan Birth Defects Clinical Research Center, West China Second University Hospital, Sichuan University, Chengdu 610000, China

**Keywords:** primary ciliary dyskinesia, pneumonia, inflammation, immunology, pathogen, imaging examination

## Abstract

Objective: This study explored the clinical features and immune responses of children with primary ciliary dyskinesia (PCD) during pneumonia episodes. Methods: The 61 children with PCD who were admitted to hospital because of pneumonia were retrospectively enrolled into this study between April 2017 and August 2022. A total of 61 children with pneumonia but without chronic diseases were enrolled as the control group. The clinical characteristics, levels of inflammatory indicators, pathogens, and imaging features of the lungs were compared between the two groups. Results: The PCD group had higher levels of lymphocytes (42.80% versus 36.00%, *p* = 0.029) and eosinophils (2.40% versus 1.25%, *p* = 0.020), but lower neutrophil counts (3.99 versus 5.75 × 10^9^/L, *p* = 0.011), percentages of neutrophils (46.39% versus 54.24%, *p* = 0.014), CRP (0.40 versus 4.20 mg/L, *p* < 0.001) and fibrinogen (257.50 versus 338.00 mg/dL, *p* = 0.010) levels. Children with PCD and children without chronic diseases were both most commonly infected with Mycoplasma pneumoniae (24.6% versus 51.9%). Children with PCD had significantly more common imaging features, including mucous plugging (*p* = 0.042), emphysema (*p* = 0.007), bronchiectasis (*p* < 0.001), mosaic attenuation (*p* = 0.012), interstitial inflammation (*p* = 0.015), and sinusitis (*p* < 0.001). Conclusion: PCD is linked to immune system impairment, which significantly contributes to our understanding of the pathophysiology of this entity.

## 1. Introduction

Primary ciliary dyskinesia (PCD) is a rare genetic disorder affecting upper and lower airway motile cilia, causing recurrent chronic respiratory infections, starting in infancy [1]. So far, the incidence rate of PCD in children is between 1/20,000 and 1/10,000 [2]. The prevalence of PCD in children with recurrent respiratory infections is up to 5%, and in children with bronchiectasis, it is estimated to be 26% [3,4]. Unexplained respiratory distress syndrome in newborns and frequent respiratory infections in children can present in PCD [5]. Early embryonic development, and the brain and genital systems can also be involved, manifesting as situs inversus, hydrocephalus, and infertility [5]. The quality of life of PCD patients is significantly lower than that of healthy individuals [6,7].

The need for early diagnosis is underscored by data showing that delayed diagnosis is linked to poorer lung function and quality of life [8]. However, there is no independent gold-standard diagnostic test for PCD [5]. Specific PCD-causative genes can cause different clinical manifestations and disease severity, which makes PCD a very distinct disease and increases the difficulty of diagnosis [9,10]. Nearly 37% of patients showed PCD-related symptoms but could not be diagnosed with PCD according to the recommended diagnostic guidelines [11]. Nasal nitric oxide measurement (nNO), transmission electron microscopy (TEM), and gene testing are used to diagnose PCD, most of which are expensive or unavailable [12,13]. As a relatively cheap and non-invasive method, nNO has a sensitivity rate of 98% and a specificity rate of 99% in detecting PCD. However, this test requires a high degree of cooperation and is usually only suitable for children over 5 years of age [13]. Moreover, approximately only 70% of individuals with clinical manifestations of PCD have known ultrastructure defects or mutations [1]. In addition, the current treatment strategy for PCD mainly refers to cystic fibrosis, a similar chronic respiratory disease, mainly focusing on promoting the airway clearance of mucus and anti-infection treatments [14]. According to a recent multicenter study, azithromycin is now the only evidence-based medication available that can reduce the aggravation of respiratory symptoms [15]. These apparent issues in diagnosis and treatment reflect the fact that physicians lack an appropriate understanding of this disease.

Motile cilia are responsible for beating rhythmically and sweeping out fluid, mucus, and pathogenic particles, which are important for mucosal defense in the respiratory tracts [1]. Recurrent respiratory infections in children with PCD are currently thought to be due to decreased ciliary clearance [16,17,18]. Recurrent respiratory infection symptoms in PCD patients include coughing, sputum production, nasal congestion, and rhinorrhea, which are non-specific compared with ordinary pneumonia [19]. However, previous studies suggest that PCD patients have specific alterations in inflammatory indicators. According to prior research, PCD patients have higher neutrophil counts, neutrophil elastase levels, and CXCL8/IL-8 levels in their sputum than healthy individuals [20]. The activity of neutrophils extracted from PCD patients is reduced, which may be crucial in the occurrence of recurrent respiratory infections [21,22,23]. The count and percentage of neutrophils and other immune cells in the blood could directly reflect the level of immune response. However, most previous studies have focused on the levels of immune cells or cytokines in the sputum, which only reflect the local immune response of the airway. Few clinical studies have focused on the composition of peripheral blood immune cells in PCD patients or explored the differences in clinical manifestations and systematic immune responses between healthy children and children with PCD during the early stages of acute infection.

Thus, we performed this retrospective study to explore the differences in clinical features and immune responses between children with and without PCD during pneumonia episodes. These findings should improve our comprehension of the clinical characteristics of PCD in the acute early stages of infection. In addition, the discovery of PCD-related immune responses may guide the development of therapeutic treatments for PCD.

## 2. Materials and Methods

### 2.1. Study Design

#### 2.1.1. Patient Enrollment

In this single-center retrospective study, we reviewed the records of children with pneumonia and unexplained recurrent respiratory infections between April 2017 and August 2022 who met the following criteria: (1) presented with two or more of the four highly suspected PCD clinical manifestations, including unexplained neonatal respiratory distress during the neonatal period, year-round daily cough starting before the age of 6 months, year-round daily nasal congestion starting before the age of 6 months, or organ laterality defects, in accordance with the ATS PCD screening criteria [24]; or (2) their score was greater than 5 points on the PICADAR scoring scale [25].

To provide a reference for assessing immune responses in children with PCD, we matched the enrolled children with PCD to control group children who suffered pneumonia at our institution during the same period. Control patients were randomly selected and matched to children with PCD at a ratio of 1:1. Age, sex, and severity of pneumonia were matched accordingly, in order to reduce bias. Children with pneumonia treated in our hospital were included according to the following criteria: (1) no history of recurrent respiratory infections; and (2) no history of chronic disease. 

Children with congenital immunodeficiency, cystic fibrosis, idiopathic pulmonary fibrosis, other airway and lung developmental malformations, foreign body aspiration, or chronic heart, liver, kidney, and blood system diseases were excluded. All children met the diagnostic criteria and hospitalization criteria for community-acquired pneumonia according to the British Thoracic Society guidelines [26]. Additionally, a PCD exacerbation was deemed to exist if three or more of the subsequent seven conditions were present, in accordance with expert consensus [27]: (1) increased coughing, (2) altered sputum volume and/or color, (3) increased shortness of breath perceived by the patient or parent, (4) decision to begin or alter antibiotic therapy due to perceived pulmonary symptoms, (5) malaise, tiredness, fatigue, or lethargy, (6) new or increased haemoptysis, or (7) temperature > 38 °C.

#### 2.1.2. Criteria for Diagnosing PCD

PCD can be diagnosed in children with highly suspected PCD who meet one of the following three conditions: low nasal nitric oxide level after excluding CF, PCD-associated genes with biallelic pathogenic variations, or a recognized ciliary ultrastructural defect [13].

### 2.2. Data Extraction

The following data for each patient was obtained from electronic medical records: age, gender, body mass index, clinical diagnosis, symptoms, lung function, TEM, results of laboratory tests, chest and nasopharynx computed tomography, and respiratory secretion bacterial culture results. The primary outcomes were differences in immune-related indicators including white blood cells (WBC), neutrophils, monocytes, lymphocytes, eosinophils, C-reactive proteins (CRPs), and fibrinogen from peripheral blood. Clinical features, pathogens, and imaging features of the PCD and control groups constituted the secondary outcome.

### 2.3. Laboratory Evaluations

Our hospital has a standardized admission process and strict requirements for the timing of blood collection for children. All of the children were admitted to the hospital within 48 h of onset. The nurse collected the venous blood on the day of the child’s admission, and sent it to the Laboratory Department of our hospital within 2 h for analysis of the blood routine examination, CRP levels, and other indicators. Immune cell counts and percentages in the peripheral blood were used to assess the systemic immune status of the PCD. CRP and fibrinogen levels were used to assess the level of inflammation. Respiratory secretions were collected, including sputum and nasopharyngeal swabs, and sent for microbial testing within 30 min. Sputum cultures were used to identify bacterial and fungal infections. Seven patients in the control group had completed etiological examinations in other hospitals before admission, and their family members refused to repeat the tests after admission. In order to ensure consistency in the test results, we performed statistical analysis after excluding patients who had completed examinations from other hospitals. An alveolar lavage fluid examination was performed for all PCD patients but only for some control patients, so this part of the data was not selected. The virus was detected in the nucleic acid of the nasopharyngeal swabs from the patients’ respiratory tracts. Serological methods were relied upon to detect atypical pathogens, namely Mycoplasma pneumoniae (MP) and Chlamydia pneumoniae (CP). Serum IgM antibody titers ≥1:160 were used to detect MP infections, and serum IgM antibody titers ≥1:32 were used to detect CP infections. Computed tomography of the chest and nasopharynx were completed within 48 h of admission for all patients. All CT scans were assessed by a radiologist with eight years of clinical experience reading chest CT scans (SD). Children who did not meet the above conditions were excluded. 

### 2.4. Ethics

The Ethical Review Board of the West China Second University Hospital, Sichuan University, granted consent for this study (approval number: 2022YFS076). All participants or their legal guardians gave their informed permission. 

### 2.5. Statistical Analyses

Continuous variables were presented as means and standard deviations (SDs) for data with a normal distribution, while categorical variables were presented as frequencies (percentages). If data were normally distributed, inter-group differences in continuous variables were assessed for significance using the independent-samples *t*-test. If the data were skewed or had an uneven variance, the Mann–Whitney U test was applied. Differences in categorical variables were assessed using the chi-squared and Fisher’s exact tests. *p* < 0.05 was considered statistically significant. All statistical analyses were performed using SPSS 23.0 (IBM, Armonk, NY, USA).

## 3. Results

**Figure 1:**
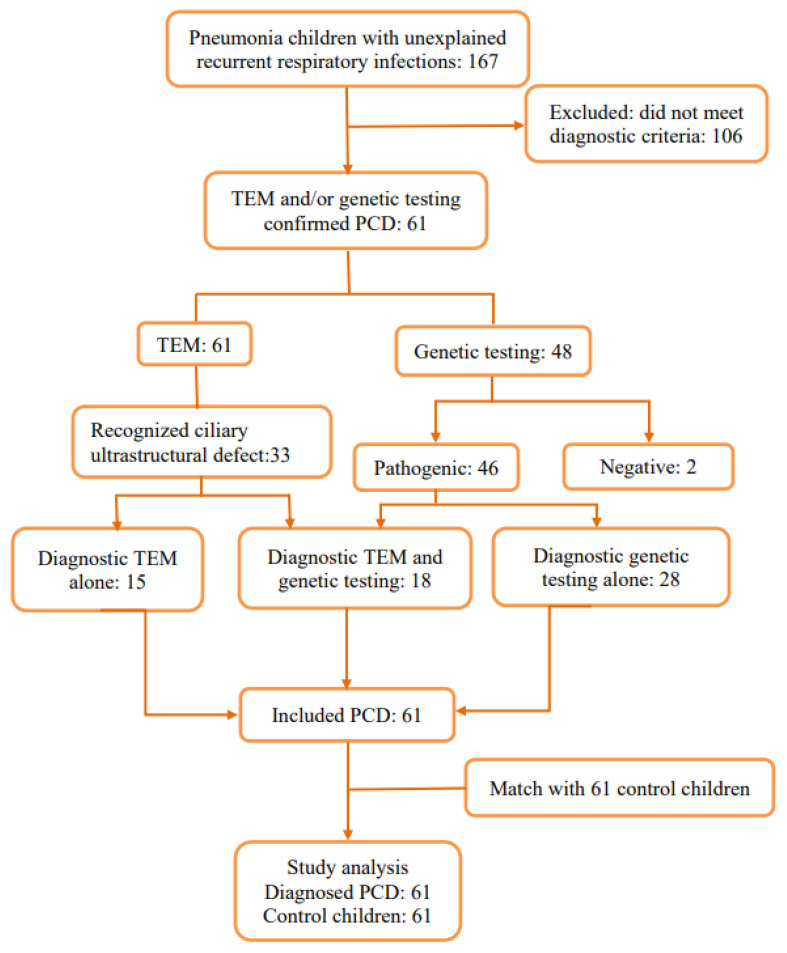
Flowchart of patient enrollment.

A total of 167 children met the PCD screening criteria of the ATS guidelines or had a PICADAR score > 5 points. From this, a further 106 children were excluded, as due to inadequate diagnostic facilities and clinician training, they had an insufficient diagnostic basis and failed to meet the diagnostic criteria. A total of 15 children were confirmed to have PCD through transmission electron microscopy (TEM), 18 children were confirmed to have PCD through TEM and genetic testing, and 28 children without recognized ciliary ultrastructural defect were confirmed to have PCD through genetic testing. In total, 61 children with PCD and 61 control children were enrolled in this study (Figure 1). 

### 3.1. Patient Characteristics

**Figure 2:**
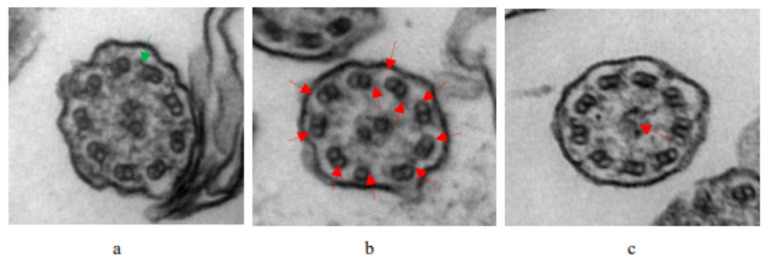
TEM images of the ultrastructure of the ciliary axonemes from a normal child and patients with PCD: (**a**) Normal ciliary axonemes in a healthy individual, indicated by the green arrow. (**b**) The lack of ODA and IDA in the patient, indicated by the red arrows. (**c**) The lack of CP in the patient, indicated by the red arrows.

Of all the patients with PCD, 32 (52.46%) were male and 29 (47.54%) were female. There were no statistical differences in gender, age, height, weight, or body mass index (BMI). Common respiratory symptoms, such as coughing, expectoration, gasping, wheezing, stuffy nose, and running nose, did not show distinctly different patterns between the PCD group and the control group. A total of 6.56% of children with PCD (4/61) had congenital heart defects (atrial septal defect, patent ductus arteriosus, and patent foramen ovale) and 11% (7/61) had asthma. Impaired lung function in PCD manifested as a decrease in FEV1% (ratio of forced expiratory volume in one second, actual measured to predicted values; mean value < 80%), FEV1/FVC% (ratio of FEV1 to forced vital capacity, actual measured to predicted values; mean value < 92%), FEV1/VC MAX% (ratio of FEV1 to max vital capacity, actual measured to predicted values; mean value < 92%), and MMEF (maximal midexpiratory flow curve, actual measured to predicted values; mean value < 65%), a combination of obstructive and restrictive ventilatory dysfunctions, and small airway dysfunction. All children with PCD underwent TEM when disease stable: eleven cases (18.03%) were found not to have abnormal cilia structures, thirty-three cases (54.10%) had typical PCD ultrastructure defects (thirty with outer dynein arm and inner dynein arm deficiency, three with the absence of a central pair), and seventeen cases (27.87%) had unidentifiable cilia structures or were difficult to classify (Table 1). To illustrate these findings, the TEM images of a child with a normal ultrastructure of the ciliary axonemes and two patients with typical PCD ultrastructure deficiency (outer dynein arm and inner dynein arm deficiency, and central pair complex abnormalities) are given in Figure 2. 

### 3.2. Inflammatory Factors

The neutrophil counts, percentages of neutrophils, and CRP and fibrinogen levels in the control group were significantly higher than those in the PCD group. Children with PCD had high percentages of lymphocytes as well as high percentages of eosinophils. The white blood cell counts, monocyte levels, and percentages of monocytes in the control group were higher than in the PCD group, while the lymphocyte and eosinophil levels were higher in the PCD group, although there were no statistical differences (Table 2). 

### 3.3. Prevalence of Respiratory Pathogens

The prevalence of bacteria differed significantly between the children with PCD and the control group children. The children with PCD and the control group children were both more commonly infected with *Haemophilus influenzae* among all kinds of bacteria, without any significant differences. Although the prevalence of atypical pathogens was high in both groups, the prevalence was significantly higher in the control group children than in the children with PCD. Compared with the children with PCD, the *Mycoplasma pneumoniae* infection rate was higher in the control group. The infection rates of fungus and viruses were low in the two groups, and there were no statistical differences. However, the fungal infection rate in the PCD group tended to be higher than in the control group, and there were no fungal infections detected in the control group (Table 3). All culture-positive patients turned negative after receiving standard anti-infection treatments.

### 3.4. Imaging Features of Chest and Nasopharynx

**Figure 3:**
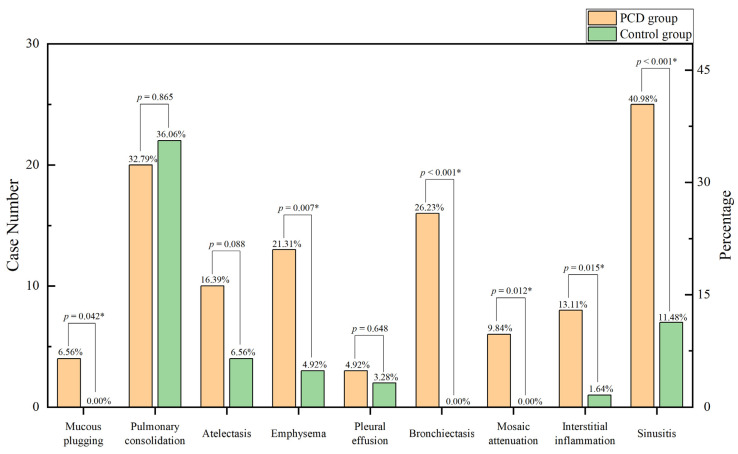
Imaging features in the two groups. * *p* < 0.05.

**Figure 4:**
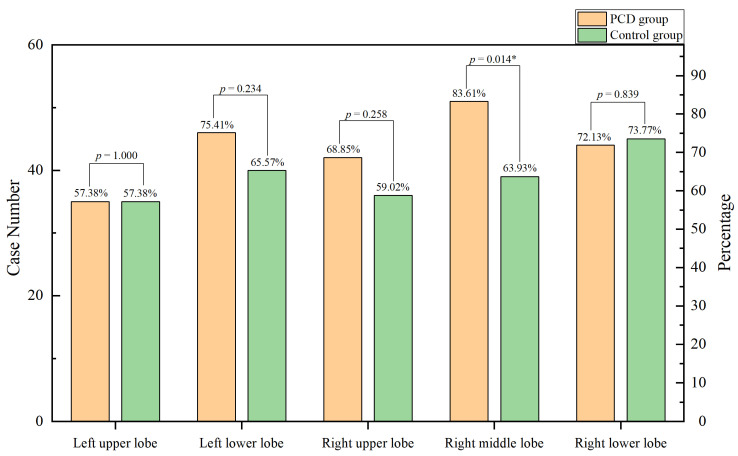
Lobe involvement in the two groups. * *p* < 0.05.

**Figure 5:**
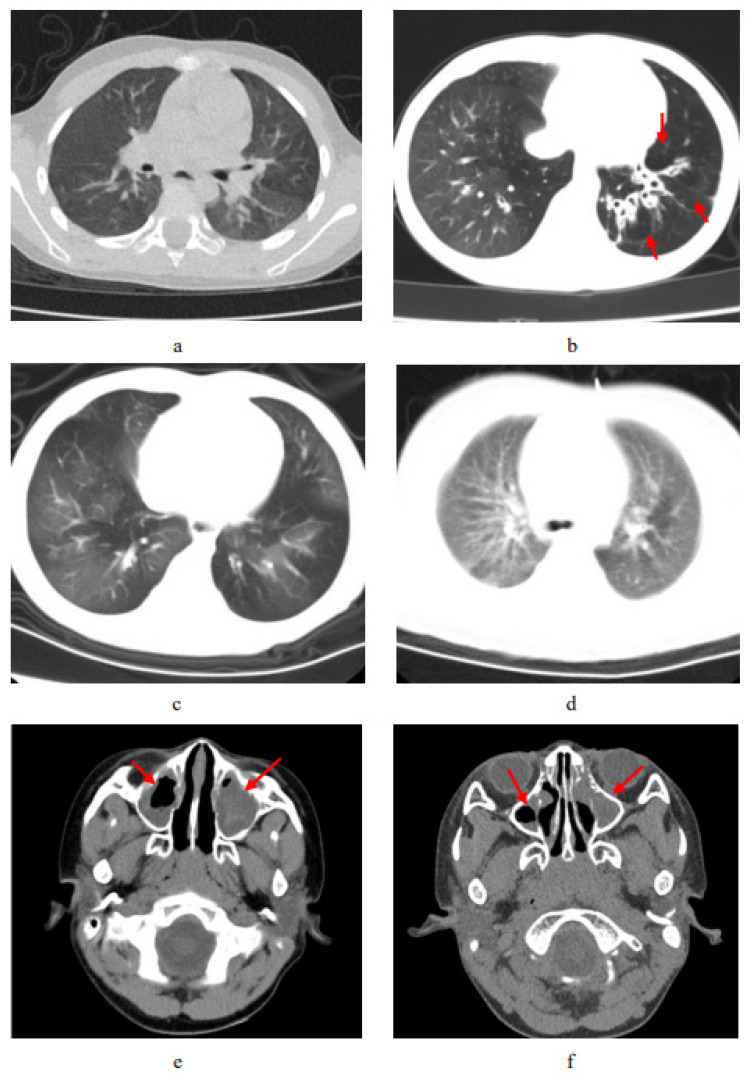
Comparison CT scan of the two groups and typical imaging features of PCD. (**a**) CT shows patchy pulmonary shadows in a control-group patient, especially in the left lobes. (**b**) Chest CT of a patient with PCD shows the bronchiectasis and compensated emphysema; the red arrow points to signs of pulmonary emphysema. (**c**) Chest CT of a patient with PCD shows interstitial inflammation. (**d**) Chest CT shows mosaic attenuation in a patient with PCD. (**e**) Nasopharynx CT shows sinusitis in a control-group patient. (**f**) Nasopharynx CT shows sinusitis in a patient with PCD. The red arrow in (**e**,**f**) points to signs of nasosinusitis.

In this study, chest computed tomography were completed for all of the children. The incidence of emphysema, bronchiectasis, mosaic attenuation, interstitial inflammation, and sinusitis was significantly increased in the PCD group. Although there were no significant differences between the two groups, the incidence of mucous plugging, pulmonary consolidation, atelectasis, and pleural effusion in the PCD group showed an increasing trend (Figure 3. The lobe most commonly infected in acute lung infections in patients with PCD was the right middle lobe, while in the control group it was the right lower lobe. The probability of right middle lobe infection in children with PCD was significantly higher than in children with pneumonia (Figure 4). Comparison CT images of the two groups and typical imaging features of children with PCD, such as emphysema, bronchiectasis, mosaic attenuation, and interstitial inflammation, have been given in Figure 5.

## 4. Discussion

This study assessed the clinical characteristics and immune responses during acute pulmonary infections in children with PCD.

In our cohort, 6.6% of children with PCD exhibited congenital heart disease (CHD), a similar frequency to that reported in previous studies. Properly working motile nodal cilia are in charge of generating a leftward flow pattern at the embryonic node, breaking the pattern of symmetry and permitting the appropriate development of internal organs, including a properly arranged, asymmetric cardiopulmonary circulation system [28]. Genetically, a higher frequency of organ laterality defects, and occasionally congenital heart disease, is linked to mutations encoding for ODA proteins, ODA plus IDA proteins, and IDA proteins with microtubule disorganization defects [29]. Moreover, many cilia-transduced cell signaling pathways, such as Shh, Wnt, Pdgf, and Tgfβ-BMP, are known to be crucial to cardiovascular development, and their disruption may contribute to the etiology of CHD [30]. A cardiac evaluation may be a good recommendation for all children with PCD. The children with PCD in our study were 5.60 years old on average, and their lungs showed mild obstructive and restrictive dysfunctions. Causes of early reduced lung function in PCD include reversible etiologies, such as transient atelectasis and sputum plug obstruction, and irreversible etiologies, such as lung remodeling following repeated severe lung infections throughout development [31]. Another important finding in our study was that a considerable proportion of clinical biopsies with electron microscopic findings showed insufficient cilia for TEM analysis; the reasons for this could be insufficient transverse sections, inadequate quality samples, or atypic polymorphous post-infection abnormalities, which appear to be a problem for the diagnosis of PCD [32,33,34]. The characteristic radiographic findings in patients with PCD included bronchiectasis, which is due to structural changes caused by recurrent respiratory infections in children. This finding is consistent with earlier studies [35,36].

We also found that the levels of neutrophils in children with PCD were lower than those in the control group children. The innate immune system is the initial line of defense against invading microbial pathogens, and plays an essential role in the early phases of infection [37]. After pathogens enter the body, airway epithelial cells or local macrophages secrete chemokines to recruit neutrophils and other immune cells from the blood to the damaged area [38]. At the local injury site, damage was exacerbated by the airway epithelial cells and macrophages, which released chemokines, and the enrichment of the neutrophils [39]. Neutrophils are the first immune cells to arrive at the site of infection, and their antimicrobial response is critical in the early stages of the inflammatory process. In our study, the decreased count and percentage of neutrophils in the blood reflected the impaired immune response of patients with PCD. Previous in vitro studies have demonstrated that blood neutrophils from patients with PCD show reduced chemotaxis to chemokines [21,22,40,41]. The chemotactic differentials in response to chemokines, including leukotriene B4, complement 5a, and N-formylmethionyl-leucyl-phenylalanine, in neutrophils from patients with PCD were substantially lower than the comparable levels in neutrophils from patients without chronic diseases [21]. Patients with PCD are prone to recurrent respiratory tract infections, which are mostly thought to be caused by ciliary dysfunction; however, these findings suggest that a weakened neutrophil immune response may also contribute. This conclusion corresponds to our results that show that children with PCD had significantly lower levels of neutrophils than the control group children. Neutrophils have a cytoplasmic microtubule system that, if abnormal and dysfunctional, can also lead to recurrent infections [41]. Our previous study found that DNAH5 mutations not only lead to impaired ciliary function, but also cause a reduced immune response [42]. We speculate that genes encoding neutrophil microstructures may overlap with PCD-related genes; more research is needed to explore and verify this. Improving neutrophil function may be a target for the future treatment of PCD. Additionally, the monocyte counts between the two groups showed no significant differences. Monocyte function is crucial for our understanding of the immune response in PCD patients. Upon stimulation with bacterial products, monocytes from PCD patients produced significantly higher levels of pro-inflammatory cytokines and chemokines compared to healthy individuals [43]. However, Walter et al. also described the normal chemotactic migration of monocytes from PCD patients [44]. Our study shows that peripheral blood monocytes in PCD patients are within the normal range, reflecting the normal chemotactic function of monocytes, which supports the findings of earlier studies. Moreover, in this study, patients with PCD had a higher percentage of eosinophils. Eosinophils are cells of the innate immune system, and are known to increase during specific immune responses, including parasite infections and allergic diseases [45]. Our research reveals that a considerable proportion of patients with PCD have coexistent asthma, which has also been reported in previous studies [36]. However, the relationship between PCD and asthma is still unclear.

Our results also found that fibrinogen and CRP levels in patients with PCD with acute respiratory infections were lower than those in control patients, which has not been reported in other studies. CRP is an acute-phase inflammatory protein that is mainly synthesized in liver cells. In the presence of calcium, CRPs can bind to polysaccharides in microbes and activate C1q to initiate the classical complement pathway of innate immunity [46]. CRPs are crucial to the inflammatory process and the host’s response to infection, involving the complement pathway, apoptosis, and phagocytosis [47,48,49]. Another indicator, fibrinogen, including thrombin and plasminogen, is demonstrated to be involved in the early stages of the innate immune system’s response, quickly walling off and eliminating encroaching invaders [50]. Fibrinogen can also potently drive acute and reparative inflammatory pathways that affect tissue damage, remodeling, and repair [51]. The decreased levels of fibrinogen and CRPs in patients with PCD may mean a decrease in innate immunity and resistance to infection, which may contribute to the increased tissue damage of these patients. This suggests that in clinical practice, fibrinogen and CRP levels may not be suitable indicators of the severity of early infection in patients with PCD.

The main pathogens that infected children with PCD were *Mycoplasma pneumoniae* (24.6%) and *Haemophilus influenzae* (19.7%), while the main pathogen infecting the control group children was *Mycoplasma pneumoniae* (51.9%). *Mycoplasma pneumoniae* infection is the most common pathogenic infection in children [52,53,54]. Interestingly, children with PCD are less susceptible to Mycoplasma pneumonia infections compared to healthy children. Mycoplasma pneumonia invades the respiratory tract, slides and moves to locate in the crypts of ciliated cells, and then adheres to receptors on the surface of respiratory epithelial cells in order to resist ciliary clearance and phagocyte engulfment, which is required for mycoplasma pneumonia to cause disease [55]. No studies have yet explored the relationship between PCD and mycoplasma pneumonia. We speculate that the lack of susceptibility in PCD patients may be caused by impaired ciliary function, which makes it difficult for mycoplasma to localize and diffuse. However, more research is needed to confirm this hypothesis. Additionally, it is noteworthy that in our study, the proportion of *Haemophilus influenzae* in patients with PCD with respiratory infections was high. The higher proportion of *Haemophilus influenza* infection in patients with PCD may be connected to the dysfunction of ciliary clearance in these patients. One explanation for the prevalence of *Haemophilus influenzae* is the development of biofilm, which cannot be effectively cleared due to the dysfunction of the ciliary [56,57]. Another explanation is the low airway NO levels in patients with PCD, which play a role in regulating the metabolic activity of these respiratory bacteria, influencing their susceptibility to antibiotics [56,57].

In contrast to previous studies on patients with PCD, we explored the relationship between PCD and immune responses during a pneumonia episode. However, we acknowledge several limitations regarding the present study. First, our study’s retrospective design may increase the potential for bias, and its relatively small sample from a single center means that our results should be interpreted with caution. Second, we did not analyze other inflammatory markers such as interleukin-6, interleukin-10, or tumor necrosis factor. Additionally, we did not do longitudinal studies on inflammatory indicators. We recommend larger, multi-center investigations to confirm and expand upon our findings.

## 5. Conclusions

Our studies showed that the neutrophil, fibrinogen, and CRP levels in children with PCD complicated by acute pneumonia infections were lower than those in the control group children, which may be related to impaired immune function and increased tissue damage. Inflammatory indicators such as fibrinogen and CRPs may not be suitable for guiding judgments on the severity of an early infection in PCD patients. The findings regarding significantly altered immune indicators greatly contribute to our understanding of the pathophysiology of this entity and may potentially provide a new perspective on PCD treatment in the future.

## Figures and Tables

**Table 1:** Characteristics of children in the PCD and control groups.

Characteristic	PCD Group (*n* = 61)	Control Group (*n* = 61)	*p*
Mean age, years (SD)	5.60 ± 3.43	5.54 ± 3.39	0.932
Female	29 (47.54%)	32 (52.46%)	0.587
Height, m (SD)	1.18 ± 0.23	1.13 ± 0.24	0.292
Z-scores for height (SD)	−0.07 ± 1.32	−0.18 ± 1.27	0.648
Weight, kg (SD)	21.55 ± 10.76	21.09 ± 11.21	0.819
Z-scores for weight (SD)	−0.003 ± 1.99	−0.08 ± 1.36	0.808
Body mass index, kg/m^2^ (SD)	16.27 ± 2.60	16.04 ± 3.29	0.712
**Symptoms**			
Cough	61 (100.00%)	61 (100.00%)	NA
Expectoration	50 (81.97%)	55 (90.16%)	0.191
Gasp	13 (21.31%)	16 (26.23%)	0.523
Wheezing	27 (44.26%)	17 (27.87%)	0.059
Stuffing nose	26 (42.62%)	20 (32.79%)	0.262
Running nose	28 (45.90%)	23 (37.70%)	0.359
Congenital heart disease	4 (6.56%)	0 (0)	0.042 *
Asthma	7 (11.00%)	0 (0)	0.006 *
**Lung function**			
FEV1 percent predicted	77.94 ± 14.14	NA	NA
FEV1/FVC percent predicted	89.67 ± 7.97	NA	NA
FEV1/VC MAX percent predicted	89.27 ± 7.85	NA	NA
MMEF percent predicted	50.31 ± 23.36	NA	NA
**TEM**			
Normal ultrastructure	11 (18.03%)	NA	NA
Fail to recognize	17 (27.87%)	NA	NA
Abnormal ultrastructure	33 (54.10%)	NA	NA
ODA and IDA deficiency	30 (90.90%)	NA	NA
CP abnormalities	3 (9.10%)	NA	NA

Values are means ± standard deviation or *n* (%), unless otherwise noted. * *p* < 0.05. NA, not application. FEV1, forced expiratory volume in one second. FEV1/FVC: ratio of FEV1 to forced vital capacity. FEV1/VC: ratio of FEV1 to vital capacity. MMEF: maximal midexpiratory flow curve. TEM, transmission electron microscopy. ODA, outer dynein arm. IDA, inner dynein arm. CP, central pair complex.

**Table 2:** Inflammatory parameters for the two children’s groups.

Parameters	PCD Group (*n* = 61)	Control Group (*n* = 61)	*p*
White blood cell, ×10^9^/L	8.39 ± 3.57	9.89 ± 4.90	0.055
Lymphocytes, ×10^9^/L	3.52 ± 2.16	3.21 ± 1.93	0.397
Percentage of lymphocytes, %	42.80 ± 17.03	36.00 ±17.09	0.029 *
Monocytes, ×10^9^/L	0.52 (0.40, 0.76)	0.60 (0.43, 0.78)	0.303
Percentage of monocytes, %	7.05 ±2.20	7.46 ±3.14	0.408
Neutrophils, ×10^9^/L	3.99 ± 2.72	5.75 ± 4.55	0.011 *
Percentage of neutrophils, %	46.39 ±17.08	54.24 ±17.76	0.014 *
Eosinophils, ×10^9^/L	0.18 (0.08, 0.34)	0.11 (0.02, 0.23)	0.059
Percentage of eosinophiles, %	2.40 (1.25, 4.70)	1.25 (0.13, 2.83)	0.020 *
C-reactive protein, mg/L	0.40 (0.40, 0.95)	4.20 (0.40, 16.50)	<0.001 *
Fibrinogen	257.50 (229.25, 289.75)	338.00 (232.00, 437.00)	0.010 *

Values are means ± standard deviation or median (interquartile range), unless otherwise noted. * *p* < 0.05.

**Table 3:** Respiratory pathogen results for the PCD and control groups.

Species	PCD Group (*n* = 61)		Control Group (*n* = 54)		*p* Value
	n	Frequency, %	n	Frequency, %	
**Bacteria**	21	34.4	9	16.7	0.030 *
***Haemophilus influenzae***	12	19.7	6	9.8	0.207
***Streptococcus pneumoniae***	2	3.3	1	1.9	0.632
***Streptococci viridans group***	2	3.3	1	1.9	0.632
***Klebsiella pneumoniae***	2	3.3	0	0	0.179
***Acinetobacter baumannii***	1	1.6	0	0	0.345
***Escherichia coli***	1	1.6	0	0	0.345
***Enterobacter cloacae***	1	1.6	0	0	0.345
***Staphylococcus aureus***	0	0	1	1.9	0.286
**Atypical pathogen**	21	34.4	31	57.4	0.013 *
***Mycoplasma pneumoniae***	15	24.6	28	51.9	0.003 *
***Chlamydia pneumoniae***	6	9.8	9	16.7	0.278
**Fungus**	4	6.6	0	0	0.055
***Candida albicans***	2	3.3	0	0	0.179
***Lodderomyces elongisporus***	1	1.6	0	0	0.345
***Candida parapsilosis***	1	1.6	0	0	0.345
**Virus**	3	4.9	6	9.8	0.217
***Influenza A virus***	1	1.6	0	0	0.345
***Rhinovirus***	1	1.6	3	5.6	0.253
***Human adenovirus***	1	1.6	1	1.9	0.931
***Bocavirus***	0	0	2	3.7	0.124

* *p* < 0.05.

## Data Availability

The datasets used and/or analyzed during the current study are available from the corresponding author on reasonable request.
